# Asepsis Versus Antisepsis

**Published:** 1886-10

**Authors:** 


### ASEFSIS VERSUS ANTISEPSIS.

The controversy now going on between the advocates of simple "cleanliness" on the one hand, and, on the other, of those who rely upon such antiseptics as corrosive sublimate, carbolic acid and iodoform, continues to grow stronger as time passes. It becomes even acrimonious. Like many other discussions of a similar nature, a proper definition of the terms used, and a comparison of the points of similarity in the two methods, would reveal the fact that both are in harmony with well established biological facts.

Cleanliness is defined by Worcester to be "freedom from dirt." If we turn to "dirt" we find it defined as "filth; mud; mire; anything that renders unclean." It is evident, from even a superficial acquaintance with the discussion, that "cleanliness" is not used in the common acceptation of the term. It is rather difficult to determine in a given case just what kind of "dirt" the reporter thinks is excluded by "cleanliness"; in one case it is germs, bacteria, etc., with their potent and multiplying power; in another, "septic matter"; in a third, "dead matter," in fact any substance, organic or inorganic, may come under this comprehensive term. The great ovariotomist, Lawson Tait, if correctly reported by a correspondent, does not class bacilli as dirt, because he says he would gladly use them as a dressing if they were sufficiently dry and absorbent. It ought to be possible for him to secure some of the *bacillus anthracis* sufficiently desiccated for his purpose. On the other hand, the antisepticists pin their faith to spray, solutions, and other means of destroying the omnipresent germ. They look upon the first class as men too conservative to ever get a new idea, and accuse them of secretly holding to the "tape-worm diathesis" of the fathers.

An examination of the two methods suggests the secret of the success of each. In the first instance, the germ is excluded, and is analogous to the experiments of Tyndall and Pasteur, in which the former exposed putrescible fluids in chambers, the sides and bottoms of which were coated with glycerine and the contained air had deposited its foreign matter; the latter, who obtained similar conditions in the catacombs of Paris and the summit of the Righi Kulm. In both cases organic matter was exposed to "clean" air without any infection, as has been proved over and over again. Similar conditions might be obtained by having an operating room constructed with only one opening for the ingress of air. By suitable filtering apparatus the air could be deprived of all foreign matter, and that already in the room would in time deposit all its floating matter, which could be retained by a sticky coating applied to the walls and floors. All instruments could be disinfected by heat, and the water used for preparing the operator, the patient, assistants, and for irrigating the wound could be sterilized by discontinuous heating. The principle of "cleanliness" here laid down ought to secure absolute immunity from wound infection. Whether it is practicable in application remains to be seen.

The principal fact thus far brought out in the discussion is that wounds may be exposed to *chemically pure* air and water with impunity. Dirty water and impure air may be rendered innocuous by spray of carbolic acid and corrosive sublimate. The successes by each method are about equal when both are in competent hands. The latter method is the easier of application. The term "cleanliness" might be dropped and "asepsis" substituted to designate those methods which endeavor to secure absence of germs. "Antisepsis" might be employed in designating those methods which have for their object the destruction of germs supposed to be already in or about a wound. The term "aseptic dressing" might be applied to dressings which are sterilized, and which protect the wound mechanically. The term "antiseptic dressing" might be used to designate dressings which are capable of destroying germs, and which protect both mechanically and chemically.

